# Comparison between coronal FLASH and sagittal double echo steady state MRI in detecting longitudinal cartilage thickness change by fully automated segmentation – Data from the FNIH biomarker cohort

**DOI:** 10.1016/j.ocarto.2025.100657

**Published:** 2025-08-05

**Authors:** Felix Eckstein, Akshay S. Chaudhari, David J. Hunter, Wolfgang Wirth

**Affiliations:** a Research Program for Musculoskeletal Imaging, Center for Anatomy and Cell Biology & Ludwig Boltzmann Institute of Arthritis and Rehabilitation (LBIAR), Paracelsus Medical University (PMU) Salzburg, Austria; b Chondrometrics GmbH, Freilassing, Germany; c Stanford University, Stanford, CA, USA; d Rheumatology Department, Royal North Shore Hospital, Sydney Musculoskeletal Health, Kolling Institute, University Sydney, Sydney, Australia

**Keywords:** Artificial intelligence, Automated segmentation, Cartilage thickness, Osteoarthritis progression, MR imaging

## Abstract

**Objective:**

Artificial intelligence (AI-) based automated cartilage analysis demonstrated similar sensitivity to change and only slighty inferior differentiation between radiographic progressors and non-progressors compared with manual segmentation. However, this finding was based on DESS MRI from the Osteoarthritis Initiative (OAI), whereas the vast majority of multicenter clinical trials rely on T1-weighted gradient echo (e.g. FLASH). Here we directly compare fully automated analysis of coronal FLASH vs. sagittal DESS, and vs. manually segmented DESS, in a sample with both FLASH and DESS MRI acquisitions.

**Design:**

Convolutional neural network (CNN) algorithms were trained on 86 radiographically osteoarthritic knees with manual expert segmentation of the medial and lateral femorotibial cartilages (coronal FLASH and sagittal DESS). Post-processing involved automated registration of CNN-based subchondral bone segmentation to reference areas. The models were applied to baseline and two-year follow-up MRIs of radiographic progressor and non-progressor knees in the Foundation of the NIH Biomarker sample of the OAI.

**Results:**

Of the 322 FNIH knees with both FLASH and DESS; 157 were radiographic progressors. Sensitivity to medial femorotibial cartilage thickness change (standardized response mean) in the progressor subcohort was −0.81 for FLASH (automated analysis), −0.74 for automatically, and −0.72 for manually segmented DESS. Differentiation from non-progressors (Cohen's D) was −0.82. −0.70, and −0.87, respectively.

**Conclusions:**

Fully automated, AI-based cartilage segmentation with advanced post-processing reveals that coronal FLASH is at least as discriminative between radiographic progressor vs. non-progressor knees as sagittal DESS MRI. Yet, performance of fully automated segmentation is slightly inferior to manual analysis with expert quality control.

**Trial id:**

Clinicaltrials.gov identification: NCT00080171.

## 1 Introduction

Osteoarthritis (OA) treatments that extend beyond symptomatic amelioration by modifying the natural history of the disease are an unmet clinical need. Quantitative morphometric assessment of joint tissue structures using 3D MRI had an important impact on the conduct of clinical trials on potential disease-modifying OA drugs over the past decades (1). Longitudinal cartilage thickness change is now being increasingly used as primary structural endpoint for potential regulatory approval[[1], [2], [3], [4], [5], [6]]. Whereas instruments that rely on manual segmentation of articular cartilage have gained medical device product status, manual segmentation can become a rate-limiting step when more and larger trials are implemented, preventing this powerful methodology from being scalable to wider application [1]. Automated segmentation of cartilage has been a topic of high interest for decades [1,[7], [8], [9]] and artificial intelligence (AI-) based methodologies have recently facilitated MRI-based cartilage analysis [[10], [11], [12], [13], [14]]. Previously, we have used a deep-learning (DL) method, applying convolutional neural networks (CNN): We showed that femorotibial cartilage thickness can be determined accurately using these automated methods, both in healthy knees [15], and in those with different grades of radiographic OA [16]. Moreover, the method displayed similar sensitivity to change and differentiation between progressors and non-progressors compared with manual expert segmentation in a pilot longitudinal application [17]. The algorithm has recently been improved to more accurately segment denuded areas of subchondral bone and cartilage morphology in the presences of full thickness loss, as often observed with more severe osteoarthritis [18].

The above longitudinal (automated) biomarker qualification study [17] was conducted on sagittal double echo steady state (DESS) MRI, acquired in both knees of the Osteoarthritis Initiative (OAI) cohort [[19], [20], [21]]. The DESS is a compelling MR image acquisition technique that provides a combined (T1-and T2-weighted) contrast, well suited for measuring cartilage thickness [22,23]. Yet, clinical DMOAD trials are most often conducted using spoiled gradient echo MRI with T1-weighted contrast, such as fast low angle shot (FLASH). The reason is that this sequence is available routinely from all manufacturers [1] and displays similar contrast between different implementations, within and across scanners [24]. One study previously compared the longitudinal performance (sensitivity to change) of sagittal DESS, coronal reformats of the DESS, and coronal FLASH, relying on manual segmentation [25]. This comparison did not identify differences in the sensitivity to change between the three approaches, but was small, may have been biased due to potential reader preference, and did not contain progressor and non-progressor-subgroups to support a differentiation of longitudinal performance metrics.

In the current study, we pursued cartilage biomarker qualification by choosing a sample that not only included subjects with longitudinal change, but pre-defined progressor and non-progressors subcohorts, i.e. the FNIH Biomarker Consortium study on the Osteoarthritis Initiative (OAI) sample[[26], [27], [28], [29]]. The FNIH study allowed us to investigate the differentiation of the sensitivity to change between two externally defined strata, progressors and non-progressors, using the fully automated segmentation methodology. This was performed using two OAI MRI protocol elements, namely coronal FLASH and sagittal DESS. Such validation and qualification steps are important, because an (imaging) biomarker that exhibits near-term change and is associated with longer-term, clinically important outcomes has the potential to become a surrogate marker (endpoint) of treatment efficacy [[29], [30], [31], [32], [33], [34], [35]], which will greatly facilitate DMOAD regulatory approval.

The objective of the current study therefore was to apply a current [18] automated (AI-based) measurement algorithm to longitudinal coronal FLASH and sagittal DESS in the same knees, in order to compare the differentiation of the sensitivity to change between pre-defined progressor and non-progressor subcohorts, without any potential reader bias from the manual segmentation process [36]. As a quantitative performance metric of the sensitivity to change, we used the standardized response mean [SRM], i.e. the mean change divided by the standard deviation of the change over the observation interval.

## 2 Method

### 2.1 Study design

The study relied on the FNIH Biomarker Consortium that evaluated the association of imaging protocols to qualify (imaging) and molecular biomarkers with structural (radiographic) and symptomatic (pain) progression in knee OA [[26], [27], [28], [29]]. It exemplifies a nested case-control study that uses clinical and imaging data from the OAI [[19], [20], [21]]. Eligible participants had at least one knee with baseline Kellgren-Lawrence grade (KLG) 1–3 from central radiographic readings, baseline and 24 ​M knee radiographs and knee MRI, serum and urine specimens, and clinical outcome data[[26], [27], [28], [29]]. Fixed flexion knee radiographs were assessed for KLG and OARSI joint space narrowing grades [37]. (Medial) radiographic progression was defined by loss in minimum radiographic joint space width of ≥0.7 ​mm from baseline to 24, 36 or 48 months; knee pain was assessed using the Western Ontario McMasters (WOMAC) pain subscale, with progression defined as a persistent (≥2 time points) increase of ≥9 points on a 0–100 normalized score from baseline at 24, 36, 48 or 60 months [[26], [27], [28], [29]].

**Fig. 1:**
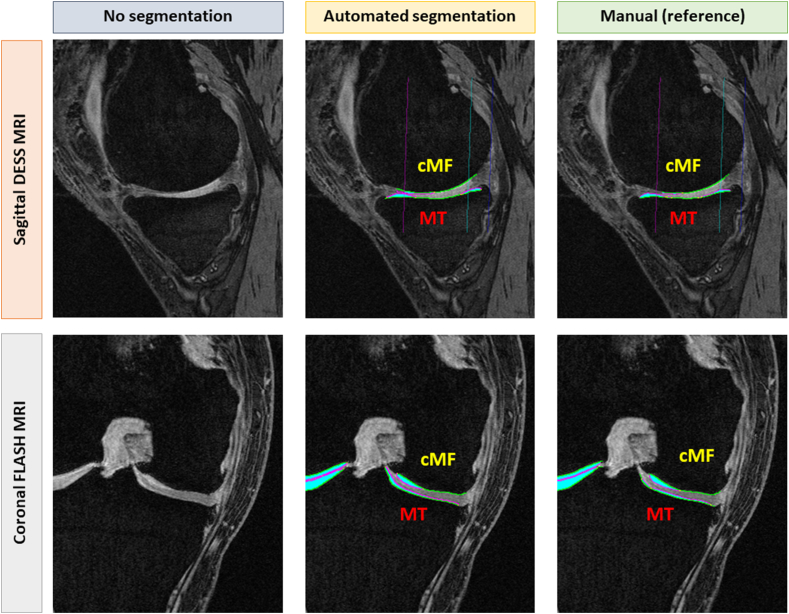
Sagittal DESS MRI (top row) and coronal FLASH (bottom row), without cartilage segmentation (left column), with automated AI-based cartilage segmentation (middle column), and with manual (reference) cartilage segmentation of the medial femoro-tibial compartment (MFTC). MT ​= ​medial tibia; cMF ​= ​weight-bearing (central) medial femur.

Cases were defined as follows: 1) primary cases included knees with both radiographic and pain progression (progressor cohort; n ​= ​194), 2) control knees with neither radiographic nor pain progression (n ​= ​200), 3) knees with radiographic but not pain progression (n ​= ​103), and 4) knees with pain but not radiographic progression (n ​= ​103)[[26], [27], [28], [29]]. For the covariates to be better balanced, the knees selected for the four groups were frequency matched, using KLG and the body mass index. Since cartilage thickness and bone shape biomarkers previously showed a strong association with radiographic but not with pain progression[[26], [27], [28], [29]], the primary comparison in this analysis was between case knees with radiographic progression (independent of whether pain progression was present or not) vs. control knees without radiographic progression (again independent of pain progression). Secondary (sensitivity) analyses were conducted for all four above groups. According to the OAI imaging protocol [19], coronal FLASH was acquired only in one (usually the right) knee to reduce the total MRI acquisition time[[19], [20], [21]]. The current analysis was hence focussed on longitudinal sensitivity to cartilage thickness change, and differentiation between progressor and non-progressor groups, in all knees that had both coronal FLASH and sagittal DESS MRI (Fig. 1). These were 322 knees, 157 radiographic (JSW) progressors, and 165 radiographic non-progressors (Table 1).

**Table 1:** Baseline characteristics of the (small) FNIH biomarker sample, i.e. knees with both sagittal DESS and coronal FLASH MRI (n ​= ​322).

N		JSW & Pain Progressors	Non Progressors	JSW Only Progressors	Pain Only Progressors
		103	108	54	57
Age	(years)	62.3 ​± ​8.8	60.9 ​± ​9.5	64.5 ​± ​7.3	60.0 ​± ​9.6
Sex	Men	44	35	25	20
	Women	59	73	29	37
BMI	(kg/m^2^)	30.7 ​± ​4.6	30.6 ​± ​4.6	30.9 ​± ​4.8	31.1 ​± ​5.2
Side	Left/right	3/100	1/107	0/54	1/56
KLG	1/2/3	12/49/42	12/65/31	8/29/17	6/36/15
med JSN	0/1/2	23/38/42	37/40/31	13/24/17	19/23/15
lat JSN	0/1	101/2	107/1	53/1	56/1
BL MFTC	DESS (manual)	3.21 ​± ​0.60	3.38 ​± ​0.50	3.34 ​± ​0.59	3.42 ​± ​0.63
(mm)	DESS (auto)	3.22 ​± ​0.56	3.35 ​± ​0.52	3.35 ​± ​0.59	3.33 ​± ​0.60
	FLASH (auto)	3.38 ​± ​0.57	3.53 ​± ​0.46	3.47 ​± ​0.57	3.49 ​± ​0.59
BL LFTC	DESS (manual)	3.90 ​± ​0.59	3.85 ​± ​0.55	3.94 ​± ​0.58	3.93 ​± ​0.61
(mm)	DESS (auto)	3.91 ​± ​0.59	3.83 ​± ​0.60	3.91 ​± ​0.56	3.88 ​± ​0.60
	FLASH (auto)	4.04 ​± ​0.54	3.95 ​± ​0.55	4.02 ​± ​0.50	3.99 ​± ​0.53

JSW ​= ​radiographic joint space width; BMI ​= ​body mass index; KLG = Kellgren Lawrence Grade; JSN ​= ​radiographic joint space narrowing; med ​= ​medial; lat ​= ​lateral, BL ​= ​baseline, MFTC ​= ​medial femorotibial compartment; LFTC ​= ​lateral femorotibial compartment; DESS ​= ​double echo steady state (MRI sequence); FLASH ​= ​fast low angle shot (MRI sequence).

### 2.2 Expert manual and automated (CNN) cartilage thickness measurement

The published femorotibial cartilage thickness segmentation of the FNIH Biomarker study relied on DESS imaging and manual expert readers (all with >15 years of experience), who were blinded to subgroup assignment and the order of image acquisition [26]. The segmentation used proprietary software (Chondrometrics GmbH, Freilassing, Germany) and included the total medial and lateral tibia (MT/LT) and the weight-bearing (central) medial and lateral femoral condyles (cMF/cLF) (Fig. 1); all segmentations were quality controlled by a PhD expert supervisor [26].

Whereas the previously published automated segmentation method on sagittal DESS [17] was almost exclusively based on a 2D U-Net CNN architecture, the current algorithm involved additional post-processing steps that allowed us to determine the (femoral) regions of interest fully automatically [18], and to increase the stability and accuracy of the subchondral bone segmentations in regions of denuded areas of bone, i.e regions with full-thickness cartilage loss, as this was a limitation of prior approaches [16,17,38].

Separate U-Nets were first trained for segmenting the cartilage surface and subchondral bone areas, respectively, for both sagittal DESS and coronal FLASH. The four models were generated from 86 expert segmented knees from the OAI with radiographic OA (KLG2/3/4 ​= ​35/34/31 ​%), for which manual segmentations of both FLASH and DESS were available (training set) [16]. None of these knees were part of the FNIH sample, and the application to the validation and test sets showed satisfactory segmentation agreement (high DICE coefficients) and thickness computation accuracy [16]. Training was performed using full-resolution, full-sized MRI slices on an NVIDIA RTX 4080TI GPU [15,16]. The training used a weighted cross entropy loss function with equal weights for each of the foreground features (i.e. the cartilages), with the background weight set to half of that used for the foreground features. The loss was minimized using Adam optimization (initial learning rate: 0.01, exponential decay rates (β_1_/β_2_) for the 1st moment estimates: 0.9/0.999); the software was implemented in Python 3.11 (Python Software Foundation, DE, USA) using the Tensorflow framework (v.2.15, Google LLC, Mountain View, CA, USA) [15].

The models were then administered to the “small” FNIH sample, i.e. the (n ​= ​322) sample that contained both DESS and FLASH MRI; the coronal FLASH model was applied to the FLASH MRIs, and the sagittal DESS model to the DESS MRIs (Fig. 1). Whereas the tibial cartilage was segmented in full, the (central) weight-bearing part of the femoral cartilage was separted from the anterior and posterior parts by automatically determining a 75 ​% ROI to the sagittal DESS (trochlear notch to posterior ends of the femoral condyles ​= ​100 ​%; Fig. 1), and a 60 ​% region of interest to the coronal FLASH [39]. Automated post-processing was applied, as mentioned previously [18], to register the CNN-based femorotibial subchondral bone area segmentation to an atlas using the Iterative Closest Point algorithm provided by the Point Cloud Library (v.1.13.1, [40]). This step allowed us to reconstruct the subchondral bone area also in cases with incomplete automated subchondral bone area segmentations from the CNNs. The atlas was derived from automated subchondral bone segmentations of the above 86 OA knees (training set) [16,18]. The cartilage segmentations were then linked to the reconstructed subchondral bone areas and automatically checked for segmentation errors such as gaps, disconnected cartilage fragments, and implausible segmentations that were corrected automatically [18]. The automated segmentations were neither quality-controlled nor manually corrected, in order to explore the “intrinsic” performance of the automated approach without any human in the loop.

Finally, the cartilage thickness was computed from the automatically segmented and post-processed contours, in the same way as from the manual ones obtained using Chondrometrics software [26]. Medial femorotibial compartment (MFTC) cartilage thickness was computed by adding thickness values in MT and cMF.

### 2.3 Statistical analysis

The primary conclusive analytic endpoint of this study was the differentiation of the longitudinal MFTC cartilage thickness loss over 24 months, in the radiographic progressor vs. non-progressor subcohorts, between coronal FLASH and sagittal DESS, using fully automated AI based cartilage segmentation. A secondary conclusive analytic endpoint was the comparison between automated and expert manual analysis in the DESS, using the smaller sample (of mostly right knees; n ​= ​322) that also contained FLASH acquisitions. Cohen's D was used as a measure of effect size, including its 95 ​% confidence interval [41], and a paired *t*-test to determine a descriptive significance level, without adjusting for multiple comparisons (SPSS 29, IBM Corporation, NY, USA). Descriptive analytic endpoints included the SRM (mean change from baseline to 24 month follow-up, divided by the standard deviation of the change over the same period) in the different subcohorts, using the different analysis methods. SRMs and 95 ​% confidence intervals were computed employing bias-corrected and accelerated bootstrapping (1000 iterations) using “R”. Finally we determined the number (proportion) of individual progressors in both cohorts, defined by published thresholds from OAI-pilot study test retest analyses [42]. The Chi square test was used to determine whether the number of MRI progressors differed significantly between the radiographic progressor and non-progressor subcohorts at a descriptive level, again without adjusting for multiple comparisons.

## 3 Results

The demographics, radiographic status, and baseline cartilage thickness properties in the medial (MFTC) and lateral femorotibial compartment (LFTC) are listed in Table 1 for the small sample (n ​= ​322; 1 knee per participant). MFTC cartilage thickness values were virtually identical between the manually vs. automatically segmented sagittal DESS in this smaller sample of knees that contained FLASH acquistions; yet, values obtained with the coronal FLASH were 0.15 ​mm greater than those derived from the sagittal DESS; this was observed across all subcohorts. These observations were also made in the LFTC; the automatically derived cartilage thickness from coronal FLASH was larger than that from sagittal DESS (Table 1).

**Fig. 2:**
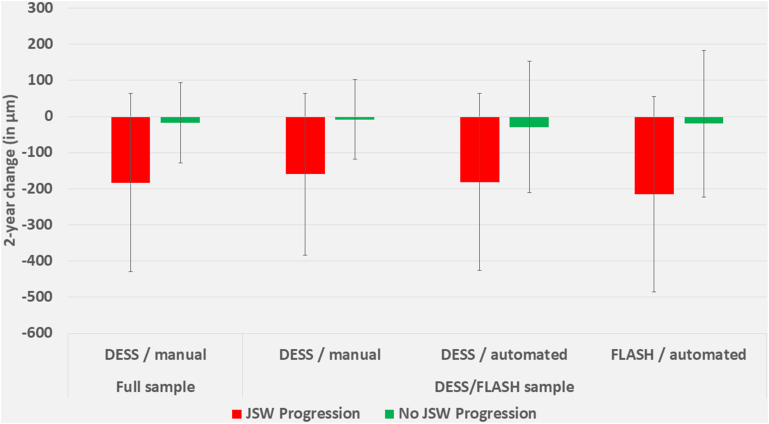
Longitudinal change in medial femorotibial compartment (MFTC) cartilage thickness between baseline and 24 month follow-up, in those with radiographic (joint space width progression {red} vs. those without {green}, for: a) The sagittal DESS MRI sequence – manual expert segmentation and quality control – in the full FNIH biomarker sample b) The sagittal DESS MRI sequence – manual expert segmentation and quality control – in the small (DESS/FLASH; n ​= ​322) sample, i.e. all knees that had both sagittal DESS and also coronal FLASH. c) The sagittal DESS MRI sequence - automated segmentation using the AI- (CNN-) based algorithm – in the small sample d) The coronal Fast Low Angle Shot (FLASH) MRI sequence - automated segmentation using the AI- (CNN-) based algorithm – in the small sample. (For interpretation of the references to colour in this figure legend, the reader is referred to the Web version of this article.)

In the MFTC, the longitudinal (24 months) cartilage thickness loss was −160 ​± ​223 ​μm in radiographic progressors vs. −8±110 ​μm in the radiographic non-progressors in the small sample (n ​= ​322) of the manual DESS, −181 ​± ​245 ​μm vs. −29 ​± ​182 ​μm for the automated DESS, and −215 ​± ​270 ​μm vs. −20 ​± ​203 ​μm for the automated FLASH (Fig. 2; Table 2). This implied an SRM of −0.72, −0.74, and −0.81 in the radiographic progressors, whereas sensitivity to change was slightly overestimated by the automated methods when using the manual analysis as ground truth (Table 2). Hence, Cohen D values of radiographic progressors vs. non-progressors were −0.87, −0.70, and −0.82, respectively. MFTC cartilage loss in the full sample (manual DESS; Fig. 2) was −183 ​± ​246 ​μm vs. −17 ​± ​111 ​μm in progressors vs non-progressors (SRM -0.75 for progressors; Cohen D −0.87 vs. non-progressors). 95 ​% confidence for the above values, and results for MT and cMF are shown in Table 2. The relative pattern of the above results in the MT and cMF was similar to MFTC, whereas the differentiation (Cohen's D) between radiographic progressors and non-progressors was greater in cMF than MT for all analyses (Table 2).

**Table 2:** Medial femorotibial compartment (MFTC) longitudinal change characteristics of radiographic (JSW) progressors and non-progressors in the FNIH biomarker sample:changes are given in μm cartilage thickness.

		JSW progression (157)	no JSW progression (165)	JSW vs. no JSW progression		
		Mean (95 ​% CI)	Mean (95 ​% CI)	Mean Diff (95 ​% CI)	Cohen's D (95 ​% CI)	% Prog
		SRM (95 ​% CI)	SRM (95 ​% CI)	P-Value		P (X^2^)
MFTC	**FLASH auto**	−215 (−258, −173)	−20 (−51, 12)	−196 (−248, −143)	−0.82 (−1.05, −0.59)	64 ​% vs. 24 ​%
		−0.81 (−0.99, −0.63)	−0.12 (−0.34, 0.04)	<0.001		<0.001
	**DESS auto**	−181 (−219, −142)	−29 (−57, −2)	−151 (−199, −104)	−0.70 (−0.93, −0.48)	56 ​% vs. 31 ​%
		−0.74 (−0.90, −0.61)	−0.17 (−0.32, −0.01)	<0.001		<0.001
	**DESS manual**	−160 (−195, −125)	−8 (−25, 9)	−152 (−191, −114)	−0.87 (−1.10, −0.64)	54 ​% vs. 19 ​%
		−0.72 (−0.85, −0.59)	−0.07 (−0.23, 0.09)	<0.001		<0.001
MT	**FLASH auto**	−64 (−85, −43)	−13 (−27, 1)	−51 (−76, −26)	−0.45 (−0.67, −0.23)	55 ​% vs. 27 ​%
		−0.49 (−0.65, −0.32)	−0.14 (−0.27, 0.01)	<0.001		<0.001
	**DESS auto**	−40 (−56, −24)	−13 (−27, 0)	−27 (−47, −6)	−0.28 (−0.50, −0.06)	32 ​% vs. 19 ​%
		−0.40 (−0.55, −0.25)	−0.15 (−0.28, 0.00)	0.012		0.007
	**DESS manual**	−41 (−55, −27)	−3 (−11, 5)	−38 (−54, −22)	−0.51 (−0.74, −0.29)	28 ​% vs. 11 ​%
		−0.45 (−0.59, −0.33)	−0.06 (−0.22, 0.10)	<0.001		<0.001
cMF	**FLASH auto**	−151 (−181, −122)	−7 (−33, 20)	−144 (−184, −105)	−0.80 (−1.03, −0.57)	62 ​% vs. 19 ​%
		−0.81 (−0.95, −0.67)	−0.10 (−0.39, 0.08)	<0.001		<0.001
	**DESS auto**	−141 (−170, −112)	−16 (−35, 3)	−125 (−159, −90)	−0.80 (−1.02, −0.57)	54 ​% vs. 30 ​%
		−0.77 (−0.92, −0.62)	−0.13 (−0.29, 0.01)	<0.001		<0.001
	**DESS manual**	−119 (−145, −93)	−5 (−17, 8)	−114 (−143, −86)	−0.89 (−1.12, −0.66)	45 ​% vs. 16 ​%
		−0.73 (−0.85, −0.61)	−0.06 (−0.20, 0.10)	<0.001		<0.001

Diff ​= ​difference; CI ​= ​confidence interval; SRM ​= ​standardized response mean; MT ​= ​medial tibia; cMF ​= ​weight-bearing medial femur; other abbreviations please see Table 1.

The number of MRI cartilage thickness progressors varied between 54 ​% (manual DESS) and 64 ​% (auto FLASH) in the radiographic progressors vs. 19 ​% (manual DESS) and 31 ​% (auto FLASH) in the non-progressors; all differences in these percent values were highly statistically significant (Table 2). Results in the LFTC did not reveal any obvious differences in longitudinal cartilage thickness changes between the above methods (Table 3).

**Table 3:** Lateral femorotibial compartment (MFTC) longitudinal change characteristics of radiographic (JSW) progressors and non-progressors in the FNIH biomarker sample:changes are given in μm cartilage thickness.

		JSW progression (157)	no JSW progression (165)	JSW vs. no JSW progression		
		Mean (95 ​% CI)	Mean (95 ​% CI)	Mean Diff (95 ​% CI)	Cohen's D (95 ​% CI)	% Prog
		SRM (95 ​% CI)	SRM (95 ​% CI)	P-Value		P (X^2^)
LFTC	**FLASH auto**	−24 (−57, 8)	−19 (−41, 2)	−5 (−43, 33)	−0.03 (−0.25, 0.19)	29 ​% vs. 24 ​%
		−0.12 (−0.25, 0.05)	−0.14 (−0.28, 0.01)	0.795		0.368
	**DESS auto**	−31 (−60, −3)	−36 (−59, −13)	5 (−32, 41)	0.03 (−0.19, 0.25)	25 ​% vs. 24 ​%
		−0.18 (−0.35, −0.01)	−0.24 (−0.39, −0.09)	0.794		0.801
	**DESS manual**	−16 (−36, 5)	−18 (−34, −2)	2 (−24, 28)	0.02 (−0.20, 0.24)	15 ​% vs. 16 ​%
		−0.12 (−0.29, 0.04)	−0.18 (−0.32, −0.03)	0.857		0.671
LT	**FLASH auto**	−27 (−51, −3)	−17 (−32, −2)	−10 (−38, 18)	−0.08 (−0.29, 0.14)	43 ​% vs. 43 ​%
		−0.18 (−0.30, −0.04)	−0.17 (−0.33, −0.02)	0.496		0.949
	**DESS auto**	−28 (−43, −12)	−37 (−48, −25)	9 (−10, 28)	0.10 (−0.11, 0.32)	25 ​% vs. 28 ​%
		−0.30 (−0.51, −0.11)	−0.50 (−0.66, −0.35)	0.349		0.536
	**DESS manual**	−20 (−30, −10)	−23 (−33, −13)	3 (−11, 17)	0.05 (−0.17, 0.26)	20 ​% vs. 18 ​%
		−0.31 (−0.49, −0.14)	−0.36 (−0.51, −0.23)	0.682		0.521
cLF	**FLASH auto**	2 (−11, 16)	−2 (−14, 9)	5 (−13, 22)	0.06 (−0.16, 0.28)	11 ​% vs. 15 ​%
		0.02 (−0.13, 0.18)	−0.03 (−0.20, 0.13)	0.604		0.317
	**DESS auto**	−4 (−22, 14)	0 (−16, 16)	−4 (−28, 20)	−0.04 (−0.26, 0.18)	12 ​% vs. 12 ​%
		−0.03 (−0.17, 0.14)	0.00 (−0.15, 0.16)	0.731		0.996
	**DESS manual**	4 (−9, 17)	5 (−5, 15)	−1 (−17, 16)	−0.01 (−0.23, 0.21)	8 ​% vs. 4 ​%
		0.05 (−0.11, 0.21)	0.07 (−0.08, 0.23)	0.945		0.195

LT ​= ​lateral tibia; cLF ​= ​weight-bearing lateral femur; other abbreviations please see Table 1, Table 2.

Table 4 displays the MFTC cartilage thickness changes in the four (sub-)cohorts, confirming that knees with radiographic (but not pain) progression (isolated joint space width progressors) behave similarly to those with combined joint space width and pain progression, and those with only pain progression similarly to those without any (JSW or pain) progression. Results in the LFTC did not reveal any obvious differences in longitudinal cartilage thickness changes between the above subgroups (data not shown).

**Table 4:** Medial femorotibial compartment (MFTC) longitudinal change characteristics of combined progressors and non-progresssors as well as partial progressors (JSW only or pain only) in the FNIH biomarker sample: changes are given in μm cartilage thickness.

		JSW & Pain (103)	None (108)	JSW only (54)	Pain only (57)
		Mean (95 ​% CI)	Mean (95 ​% CI)	Mean (95 ​% CI)	Mean (95 ​% CI)
		SRM (95 ​% CI)	SRM (95 ​% CI)	SRM (95 ​% CI)	SRM (95 ​% CI)
MFTC	**FLASH auto**	−207 (−258, −155)	−38 (−64, −11)	−232 (−309, −154)	14 (−62, 90)
		−0.79 (−1.06, −0.55)	−0.28 (−0.47, −0.09)	−0.84 (−1.12, −0.63)	0.01 (−0.33, 0.23)
	**DESS auto**	−174 (−219, −128)	−51 (−83, −18)	−195 (−268, −122)	11 (−41, 63)
		−0.74 (−0.95, −0.56)	−0.30 (−0.47, −0.12)	−0.74 (−0.95, −0.54)	0.06 (−0.20, 0.31)
	**DESS manual**	−154 (−201, −108)	−9 (−29, 12)	−170 (−224, −116)	−6 (−37, 25)
		−0.66 (−0.82, −0.51)	−0.08 (−0.26, 0.13)	−0.88 (−1.14, −0.66)	−0.05 (−0.32, 0.21)
MT	**FLASH auto**	−74 (−102, −46)	−16 (−35, 2)	−45 (−75, −15)	−6 (−29, 17)
		−0.53 (−0.74, −0.34)	−0.17 (−0.33, 0.00)	−0.43 (−0.74, −0.15)	−0.07 (−0.34, 0.20)
	**DESS auto**	−36 (−55, −16)	−16 (−32, −1)	−48 (−76, −20)	−7 (−34, 20)
		−0.36 (−0.56, −0.16)	−0.21 (−0.37, −0.02)	−0.48 (−0.70, −0.28)	−0.06 (−0.30, 0.21)
	**DESS manual**	−40 (−58, −21)	−4 (−14, 5)	−43 (−66, −20)	−1 (−15, 14)
		−0.43 (−0.58, −0.28)	−0.09 (−0.28, 0.11)	−0.53 (−0.78, −0.30)	−0.01 (−0.29, 0.25)
cMF	**FLASH auto**	−133 (−166, −100)	−21 (−37, −5)	−186 (−247, −126)	20 (−51, 92)
		−0.80 (−1.02, −0.59)	−0.27 (−0.53, −0.04)	−0.87 (−1.08, −0.68)	0.01 (−0.39, 0.23)
	**DESS auto**	−138 (−173, −103)	−34 (−57, −12)	−147 (−201, −92)	18 (−16, 52)
		−0.79 (−1.02, −0.61)	−0.30 (−0.49, −0.11)	−0.74 (−0.96, −0.53)	0.14 (−0.13, 0.41)
	**DESS manual**	−115 (−147, −82)	−4 (−19, 11)	−128 (−171, −84)	−5 (−29, 19)
		−0.69 (−0.85, −0.55)	−0.05 (−0.25, 0.14)	−0.81 (−1.05, −0.58)	−0.06 (−0.33, 0.19)

Abbreviations please see Table 1, Table 2.

The computation time for the automated segmentation and post-processing was about 1 ​min per visit for all methods, and the computation of the cartilage and subchondrondral morphometry measures about another minute.

## 4 Discussion

We here examined whether a fully automated (AI-based) measurement algorithm differentiates the sensitivity to change between pre-defined progressors and non-progressors similary, using coronal FLASH (most commonly used in multi-centre clinical trials) and sagittal DESS (used primarily in the OAI), and how the contrast between these compares with that from manually segmented DESS. We find that the ability of coronal FLASH to differentiate between progressors and non-progressors is at least as high as that of sagittal DESS MRI; yet, performance of manual segmentation (DESS) is still slightly superior in differentiating progressors and non-progressors compared with automated analysis.

The FNIH OA Biomarkers Consortium study relied on OAI data and the OAI MR imaging biomarkers for predicting and/or for displaying sensitivity to concurrent change with clinically relevant strata of (medial) femorotibial OA progression [28]. Manual segmentation of MFTC cartilage demonstrated substantial loss over 24-months in the participants with (combined or isolated) radiographic progression [26]. Therefore this sample is well suited for testing the longitudinal performance of automated segmentation algorithms [17] and MRI protocol elements with different contrast and orientation [25], in their ability of capturing structural OA progression. Moreover, given that the FNIH subgroups without radiographic progression did show very little cartilage loss, the study lends itself to studying the differentiation between progressor and non-progressor groups; similar to a putative clinical trial where the placebo group is progressing, whilst the investigational medical product is expected to ameliorate cartilage loss.

We here applied fully automated cartilage analysis (without expert intervention or QC) using two typical cartilage morphometry protocol elements, one (FLASH), most commonly used in multi-centre clinical trials, and one (DESS) spear-headed by the OAI. The coronal FLASH (automated segmentation) differentiated the sensitivity to change (SRM) similarly, or even slightly better between pre-specified FNIH progressors and non-progressors, compared with automated sagittal DESS. Yet, both automated approaches slightly overestimated cartilage loss in the non-progressors vs. manual analysis (ground truth), so that manual segmentation (DESS) was still somewhat superior in differentiating progressors and non-progressors (Cohen's D) to automated analysis. Yet, the automated methodology performed as well as the manual segmentation apporoach in capturing the sensitivity to change in progressors.

CNN-based automated segmentation techniques have been shown to segment articular cartilage from MRI with relatively high accuracy, even in knees with advanced radiographic OA [16,38]. The segmentation of denuded areas of subchondral bone (dABs) with full thickness cartilage loss, is challenging [38], whereas dABs are important constructs frequently observed in advanced knee OA [43], and shown to be related to structural progression [44] and pain [45,46]. In contrast to our previous approach [17] we here used a novel algorithm that also segments the subchondral bone and applies post-processing steps to accurately capture cartilage morphology in severe OA [18]. This also increased the stability of the algorithm, so that all cases of the sample were be successfully computed without manual intervention, with rapid computation and post-processing time.

A limitation of the current paper is that only about half of the FNIH sample could be used, because the FNIH biomarker study had selected both left and right knees (depending on which one fulfilled the inclusion criteria), whereas the coronal FLASH was mostly acquired in right knees only in the OAI [[19], [20], [21]]. Whilst this may have somewhat dysbalanced the frequency matching of case and control knees, this is not crucial to the objective of the current study, as we compared the performance metrics of different protocol elements and analytic methods in identical knees.

The orientations between FLASH (coronal) and DESS (sagittal) were different, as the OAI did not provide sagittal FLASH for direct comparison [[19], [20], [21]]. FLASH has been used with coronal [2] and sagittal [5] orientation in clinical trials. DESS can be reformatted to a coronal orientation, and a previous study has reported highly similar cartilage morphology metrics and sensitivity to change over one year between sagittal DESS, coronal DESS reformats, and coronal FLASH [25]. For this reason, and because in a clinical trial one would always use the original (sagittal) DESS, we compared sagittal DESS with coronal FLASH, using the same quantitative measure (cartilage thickness in mm). Further, the femoral regions of interest differed slightly, with coronal FLASH supporting a 60 ​% (trochlear notch to the posterior end of the femoral condyles ​= ​100 ​%) and the sagittal DESS a 75 ​% ROI [25]. This may have caused the somewhat thicker baseline values seen with FLASH, since the more posterior part of the femoral cartilage included in the DESS segmentations (60 ​%→75 ​% usually being thinner than the anterior part [47]). Yet, longitudinal sensitivity to change of cartilage loss in OAI knees in the 75 ​% femoral ROI was very similar to that in a 60 ​% (sagittal) ROI [25]. Another limitation is that – opposite to DESS - no manual segmentations were available for the coronal FLASH, so no direct comparison could be made between manual and automated analysis (FLASH), but the comparison can be made indirectly through comparison with automated DESS analysis.

A strength of our work was that the qualification of the automatically generated imaging biomarkers goes much beyond demonstrating segmentation similarity (DICE coefficients) or application in a cross-sectional setting. Another strength is that the model generation, testing and validation between the FLASH and DESS [16], and testing its performance were executed in identical knees, using different elements of a typical cartilage morphometry MRI protocol. Using the automated segmentation methodology, an unbiased and direct parallel comparison could be made. This provided an optimal setting for exploring whether the FLASH, more often used in multicenter clinical trials due to its availability across most clinical scanners/vendors, displays similar ability to distinguish rates of cartilage loss in radiographic progressors vs. non-progressors as the DESS. Moreover, the automated method applied here did not require human interaction during the segmentation process (as the case for semi-automated methods), and did not require expert quality control.

As some methodological improvements were made [18], the differentiation between radiographic progressors vs. non-progressors made with automated DESS in the full sample was somewhat superior (Cohen's D ​= ​−0.74) to our previous findings (−0.68) [17] and those from other authors [10]. The results from manual analysis in the full DESS sample (−0.87) were consistent with manual analysis in the small DESS sample (−0.87). Yet, whether viewed in the full or small sample, automated analysis did not entirely reach the degree of differentiation of manual analysis. Of note though, the manual method not only involved segmentation by expert readers, but also quality control readings, with 100 ​% coverage of all segmentations by a supervisor expert. Future work may explore whether training sets, specific to age, sex, BMI, or radiographic disease stage can improve the performance of the automated analysis further to “close the gap” with manual approaches.

In conclusion, we here applied a fully automated AI-based analysis approach that includes specific post-processing steps, to capture also severely osteoarthritic cartilage. FLASH MRI is dominantly used in multi-center clinical trials due to its availability across all vendor platforms. We find coronal FLASH to distinguish at least as well between radiographic progressors and non-progressors as sagittal DESS when using unbiased, automated segmentation methods. The performance, however, fell somewhat short of that of manual analysis with expert quality control; further efforts in improving AI-based automated segmentation are therefore desirable.

## Author contributions

All authors have made substantial contributions to.

(1) the conception and design of the study, or acquisition, or analysis and interpretation of data,
(2) drafting the article or revising it critically for important intellectual content,
(3) final approval of the version to be submitted.

## Role of the funding source

The image acquisition was supported by the Osteoarthritis Initiative (OAI), a public-private partnership comprised of five contracts (N01-AR-2-2258; N01-AR-2-2259; N01-AR-2-2260; N01-AR-2-2261; N01-AR-2-2262) funded by the National Institutes of Health, a branch of the Department of Health and Human Services, and conducted by the OAI Study Investigators. Private funding partners include Pfizer, Inc.; Novartis Pharmaceuticals Corporation; Merck Research Laboratories; and GlaxoSmithKline. Private sector funding for the OAI is managed by the Foundation for the National Institutes of Health.

The manual image analysis (previously published) was funded by a vendor contract from the Foundation of the National Institute of Health (fNIH). The general development of automated methods for tissue segmentation in our group is funding by the Ludwig Bolzmann Institute of Arthritis and Rehabilitatino (LBIAR), with the current article being part of the clinical validation/qualification steps run under this program.

None of the funding sources had any influence on the study design, data analysis, or interpretation of the data; the funding sources neither approved the study nor the paper as written.

## Declaration of competing interest

• Felix Eckstein is co-owner and CEO of Chondrometrics GmbH, a company providing MR image analysis services to academic researchers and to the pharmaceutical industry. He has provided consulting services to Merck KGaA Galapagos/Servier, Kolon Tissuegene, Novartis, Peptinov, Formation Bio, 4P Pharma, Sanofi, and Artialis.
• Wolfgang Wirth has a part time employment with Chondrometrics GmbH and is a co-owner of Chondrometrics GmbH
• Akshay Chaudhari has provided consulting services to Patient Square Capital, Chondrometrics GmbH, and Elucid Bioimaging; is co-founder of Cognita; has equity interest in Cognita, Subtle Medical, LVIS Corp, Brain Key.
• David J Hunter is the Editor of the osteoarthritis section for UpToDate and co-Editor in Chief of Osteoarthritis and Cartilage. He provides consulting advice on scientific advisory boards for Haleon, TLCBio, Novartis, TissueGene, Sanofi, and Enlivex.
